# Vitamin K Deficiency Bleeding in Infancy

**DOI:** 10.3390/nu12030780

**Published:** 2020-03-16

**Authors:** Shunsuke Araki, Akira Shirahata

**Affiliations:** 1 Department of Pediatrics, School of Medicine, University of Occupational and Environmental Health, Kitakyushu 807-8555, Japan; 2 Kitakyushu Yahata Higashi Hospital, Kitakyushu 805-0061, Japan

**Keywords:** intracranial hemorrhage, PIVKA, intramuscular injection, prophylaxis

## Abstract

Vitamin K is essential for the synthesis of few coagulation factors. Infants can easily develop vitamin K deficiency owing to poor placental transfer, low vitamin K content in breast milk, and poor intestinal absorption due to immature gut flora and malabsorption. Vitamin K deficiency bleeding (VKDB) in infancy is classified according to the time of presentation: early (within 24 h), classic (within 1 week after birth), and late (between 2 week and 6 months of age). VKDB in infancy, particularly late-onset VKDB, can be life-threatening. Therefore, all infants, including newborn infants, should receive vitamin K prophylaxis. Exclusive breastfeeding and cholestasis are closely associated with this deficiency and result in late-onset VKDB. Intramuscular prophylactic injections reduce the incidence of early-onset, classic, and late-onset VKDB. However, the prophylaxis strategy has recently been inclined toward oral administration because it is easier, safer, and cheaper to administer than intramuscular injection. Several epidemiological studies have shown that vitamin K oral administration is effective in the prevention of VKDB in infancy; however, the success of oral prophylaxis depends on the protocol regimen and parent compliance. Further national surveillance and studies are warranted to reveal the optimal prophylaxis regimen in term and preterm infants.

## 1. Introduction

Vitamin K is a fat-soluble vitamin and a necessary cofactor for the synthesis and activation of coagulation factors II (prothrombin), VII, IX, and X (vitamin K-dependent coagulation factors) and proteins C and S in the liver [1]. Vitamin K plays a critical role as a cofactor of gamma-glutamyl carboxylase in the conversion of glutamic acid to gamma-carboxyglutamic acid residues in the N-terminus of vitamin K-dependent procoagulant factors and proteins C and S [2]. Three forms of vitamin K are known: vitamin K_1_ (phylloquinone), vitamin K_2_ (menaquinones), and vitamin K_3_ (menadione) [3]. Vitamin K_1_ (phylloquinone) is the major circulating form and is primarily provided by dietary sources, such as green leafy vegetables [3]. Vitamin K_2_ (menaquinones) is found in the diet, particularly in egg yolks, chicken, beef, vegetables, and fermented products, such as natto [3]. In addition, vitamin K_2_ is synthesized from the gut flora (intestinal bacteria) and scavenger receptor class B type I [4], and Niemann–Pick C1–Like 1 [5] has recently been reported as a key regulator of intestinal vitamin K absorption. Vitamin K_3_, a synthetic form, is not being used currently for intramuscular (IM) vitamin K prophylaxis in humans because of hemolytic anemia that can occur in glucose-6-phosphate dehydrogenase-deficient infants [6].

**Figure 1:**
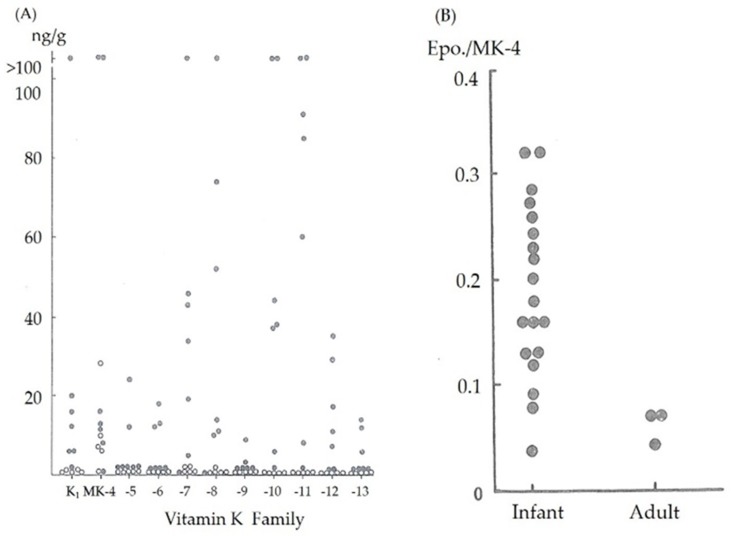
(**A**) Vitamin K (K_1_, or phylloquinone and menaquinone (MK)) content in the liver tissue in adults (closed circles) and neonates (open circles) without definite liver dysfunction. (**B**) Menaquinone-4 2, 3-epoxide/menaquinone-4 (Epo./MK-4) ratio after vitamin K (MK-4) loading in normal adults and 1-month-old infants.

Because vitamin K is available from various sources, vitamin K deficiency is a rare condition in adult humans. However, the situation differs in newborn infants because the vitamin K levels transferred from the mother to the child across the placenta are quite low. The vitamin K levels in the cord blood are often below the detection limit of 0.02 ng/mL in healthy newborns [7]. We have previously reported that vitamin K levels in umbilical blood were extremely low and that newborn infants have few vitamin K reserves in their liver tissue at birth regardless of their gestational age from 5 neonates who died within 24 h of birth after receiving no vitamin K supplements [8,9] (Figure 1A). Although breast milk is the preferred dietary mainstay for all neonates, vitamin K levels in breast milk are significantly lower than those in formula milk (median 2.5 mg/L vs. 24–175 mg/L, respectively) [10,11], and there are substantial differences in these levels among individuals [8] (Table 1).

In addition, the gut flora of neonates is immature and the amount of vitamin K synthesized from their flora is insufficient. Our previous report showed that the vitamin K content in infant fecal samples was significantly lesser than that in adult fecal samples [8] (Table 2) and supports the notion that the immature gut flora in neonates is related to vitamin K deficiency.

The reduced form of vitamin K (vitamin K hydroquinone) is oxidized to vitamin K 2, 3-epoxide, which is further reduced by vitamin K 2, 3-epoxide reductase to vitamin K. Vitamin K is then reduced by vitamin K reductase to vitamin K hydroquinone and reused. A reduction in the activity of vitamin K 2, 3-epoxide reductase results in the accumulation of vitamin K 2, 3-epoxide in liver tissues after vitamin K loading. Nishimura et al. reported a strongly significant correlation between vitamin K 2, 3-epoxide levels in the blood and liver tissue (Nishimura N, Usui Y, Kobayashi N; Menaquinone-4, vitamin K1 and epoxide levels in blood after intravenous administration of menaquinone-4 and vitamin K1. Proceedings of 5th seminar of vitamin K function. Eisai, Tokyo, pp155-162,1989). Therefore, we estimated the vitamin K 2, 3-epoxide reductase activity by measuring the ratio of menaquinone-4 2, 3-epoxide to menaquinone-4 serum levels 30 min after intravenous (IV) vitamin K administration. The menaquinone-4 2, 3-epoxide/menaquinone-4 ratio was higher in young infants than in adults [8] (Figure 1B), indicating that the activity level of vitamin K 2, 3-epoxide reductase in infants was lower than that in adults, and the reuse of vitamin K in the liver was decreased.

Carriers of the vitamin K epoxide reductase complex 1 (VKORC1) and/or cytochrome P450 2C9 (CYP2C9) variant alleles are at risk of developing vitamin K deficiency [12,13]. A frequent single nucleotide polymorphism (SNP)—G-1639A—within the VKORC1 promoter has been identified as a major determinant of coumarin sensitivity, reducing the activity of vitamin K epoxide reductase enzyme to 50% of that of the wild GG type. Polymorphisms in VKORC1 (G-1639A) and coagulation factor 7 (F7-323 Ins10) were reportedly associated with intraventricular hemorrhage or low factor VII levels [14]. Recently, the presence of variant alleles in VKORC1 (SNP: rs9923231) was reportedly associated with vitamin K deficiency bleeding (VKDB) [15]. Although reports that suggest a relationship between VKORC1 genetic variants and VKDB in Japan are lacking, the low enzyme activity of vitamin K epoxide reductase might be associated with VKDB in infancy because of the ethnic differences in the incidence of VKDB in infancy.

We summarized the possible factors inducing vitamin K deficiency in neonates (Table 3). Poor vitamin K content in breast milk does not appear to cause VKDB because we have observed that MK-4 levels in the breast milk from mothers whose children experienced idiopathic VKDB increased in a dose-dependent manner following oral MK-4 administration [8].

Therefore, vitamin K deficiency might occur if vitamin K intake during the early neonatal period is insufficient. The half-lives of vitamin K-dependent coagulation factors are short, and vitamin K deficiency may consequently lead to neonatal VKDB. Particularly, VKDB is a hemostatic disorder in which the coagulation parameters can be rapidly managed by vitamin K supplementation.

## 2. Epidemiology and Classification of VKDB

VKDB in newborns was first reported by Charles Townsend in 1894 [16]. He reported 50 neonates with a bleeding disorder that occurred during days 2–3 after birth; this disorder was termed as hemorrhagic disease of the newborn (HDN) [16] and is currently classified as the classic VKDB [17]. The term VKDB replaced the term HDN because VKDB may also occur during the postnatal period. Subsequently, several studies [18,19] have revealed that vitamin K deficiency can be a major pathophysiological factor of HDN.

Cases that are currently classified as late-onset VKDB were first reported in Thailand in 1966. Bhanchet et al. [20] summarized the cases of 93 breastfeeding Thai infants who typically presented with bleeding episodes between 1 and 2 months after birth. In Thailand, surveillance data obtained from 1981 to 1995 [21] revealed that the incidence of late-onset VKDB was higher (72 per 100,000 births) than that in European countries and that the incidence of intracranial hemorrhage was 82%. Conversely, in the first Japanese nationwide survey of vitamin K deficiency in infants conducted between 1978 and 1980, the incidence of late-onset VKDB was 8.8 per 100,000 births [22]. Therefore, even in the same Asian region, there was a significant discrepancy in the incidence of this condition.

In 1985, Lane and Hathaway summarized three types of VKDB as follows: early-onset (occurring within the first 24 h after birth), classic (occurring within days 2–7), and late-onset (occurring between 2–12 weeks and up to 6 months of age) [23] (Table 4).

Early-onset VKDB is commonly associated with maternal malabsorption disorders and medications that inhibit the activity of vitamin K, such as antiepileptics (carbamazepine, phenytoin, and barbiturates), anti-tuberculosis medications (isoniazid and rifampicin), certain antibiotics (cephalosporins), and vitamin K antagonists (warfarin); moreover, it is more common in pregnant women who do not receive vitamin K prophylaxis before delivery [24]. Recent studies have revealed that placental functions would be impaired in cases of pre-eclampsia and intrauterine growth restriction. These conditions might affect the placental transfer of vitamin K [25,26]. The incidence of early-onset VKDB in at-risk neonates who did not receive vitamin K supplementation reportedly varied from 6% to 12% [24,27,28]. Among cases that did not receive vitamin K administration before delivery, the occurrence of early-onset VKDB is frequent, and the prognosis of such infants is poor because VKDB has a high incidence of intracranial hemorrhage [29]. In 2017, the Japanese Society of Obstetrical, Gynecological, and Neonatal Hematology conducted a nationwide questionnaire survey to determine the epidemiology of early-onset VKDB between 2007 and 2016. There were 20 reported cases (2 sets of twins), including 18 fetal cases. Among the 18 mothers with early-onset and fetal VKDB infants, 11 mothers were malnourished, and the remaining had Crohn’s disease (*n* = 3) or were undergoing warfarin therapy (*n* = 4). Intracranial hemorrhage was the most common clinical feature (*n* = 12) and resulted in severe neurological sequelae (*n* = 9) and intrauterine fetal death (*n* = 2). No cases involved prophylactic vitamin K administration to the mother (unpublished data).

Conversely, classic VKDB, which is associated with low vitamin K content in breast milk, poor feeding of milk, and/or inadequate vitamin K prophylaxis, has a good prognosis because its bleeding sites are typically located in the intestinal tract. In addition, a natural decrease in the activity of prothrombin during this period has previously been reported [30,31]. Without vitamin K prophylaxis, classic VKDB is reported to occur in 0.25% to 1.7% of neonates without underlying diseases [32].

The late-onset VKDB occurs between 2 weeks and 6 months after birth, with an increased occurrence reported between 3 and 8 weeks after birth [33]. It has an incidence of 4.4 to 72.0 per 100,000 live births in Asia and Europe (Table 5).

The late-onset VKDB is classified as idiopathic without the risk factors of vitamin K deficiency except for breastfeeding or as secondary VKDB with risk factors of vitamin K deficiency, such as malabsorption or cholestasis, because vitamin K absorption closely depends on the intestinal availability of bile [3]. In the first [22] and second [34] Japanese nationwide surveys, 427 idiopathic cases and 57 secondary cases were reported, indicating that idiopathic cases are 7.5 times more common than secondary cases in Japan. Of the cases reported, 476 (87.7%) involved exclusive breastfeeding. This ratio is remarkably high compared with the mean rate of breastfeeding in Japan.

The hemorrhagic manifestations of late-onset VKDB mainly involve the gastrointestinal tract, skin, and central nervous system and present as intracranial hemorrhage. Most reported cases of late-onset VKDB have presented with intracranial hemorrhage. According to the abovementioned Japanese survey [22,34] for hemorrhagic sites, intracranial hemorrhage was reported in 82.7% of infants with idiopathic VKDB. Furthermore, intracranial hemorrhage was also significant in infants with secondary VKDB. Late-onset VKDB might affect morbidity and mortality, with the mortality being as high as 20%–50% [33]. In a Japanese survey [23,35] including 427 patients with idiopathic VKDB, 62 patients (14.5%) died and 171 (40.0%) survived with severe neurological sequelae. Interestingly, a high number of reports of late-onset VKDB, particularly idiopathic VKDB, was from South East Asia and Australia [35]. Late-onset VKDB is more frequent in the Asian population than the Caucasian population [35]. This finding may be partially explained by dietary habits or the 6-fold higher incidence of biliary atresia in Asia than in Western Europe [36]. Recent studies have confirmed that genetic variants of 10q24 are closely associated with the epidemiology of biliary atresia [37,38].

## 3. Diagnosis of VKDB in Infancy

The diagnosis of VKDB is commonly indicated by a prolonged activated partial thromboplastin time (APTT) and prothrombin time (PT). VKDB is characterized by a PT international normalized ratio (INR) ≥ 4 or a value > 4 times the normal values in the presence of normal platelet count and fibrinogen level. The diagnosis is confirmed based on the increased levels of proteins induced by vitamin K absence or antagonists (PIVKAs) and a rapid normalization of coagulation parameters, such as APTT and PT, after vitamin K administration, or both.

PIVKAs are undercarboxylated precursor proteins of vitamin K-dependent coagulation factors induced by vitamin K deficiency [39,40]. PIVKAs are released from the liver into the blood but lack calcium-binding activity and are thus inactive. PIVKAs are often measured to determine the presence of subclinical vitamin K deficiency and can be detected before the development of complications, including coagulopathies, owing to vitamin K deficiency [41,42]. PIVKA levels are correlated with the severity of deficiency, and an increase in their levels is considerably more common in breastfed infants. Vitamin K levels in the cord blood of both term and preterm infants are undetectable; however, PIVKA-II is only elevated in 10% to 50% of cord blood samples [41,43]. A previous study including term and preterm infants revealed that there was no significant relationship between gestational age and PIVKA-II levels in the cord blood [44].

Although the serum vitamin K (phylloquinone and menaquinone) level is a useful status indicator [45], its use is not practical because of the technical difficulty involved. Reference values in healthy adults range from 0.2 to 1.0 µg/L (median, ~0.5 µg/L).

Previously, we have successfully performed a screening test program to detect vitamin K deficiency in a breastfed infant population using Normotest, which can determine the activity of factors II, VII, and X using 10-μL heel puncture blood specimens collected in microtubes. This screening test program has been applied in only Japan. However, the screening test program was substituted for prophylactic vitamin K administration because heel punctures are more invasive, difficult, and expensive than vitamin K administration [46].

Several reports have revealed that genetic factors can influence the vitamin K-dependent coagulation system and intraventricular hemorrhage [14,15]. Further studies regarding the usefulness of testing for genetic variants to prevent and diagnose VKDB are warranted.

## 4. Treatment of VKDB in Infancy

There is little evidence regarding treatment for infants with VKDB. Infants with non-life-threatening bleeding should be treated with phylloquinone (vitamin K_1_; phytomenadione; phytonadione) via slow IV injection. A single IV dose of 250–300 μg/kg body weight is commonly recommended, and a dose of 1–2 mg is assumed to be sufficient to completely manage vitamin K deficiency in infants aged up to 6 months [47]. The guidelines on vitamin K administration for vitamin K deficiency in infancy, which were proposed by the Japan Pediatric Society in 2011 [48], recommend IV vitamin K_2_ (menaquinone-4, MK-4) administration, with a dose ranging from 0.3 to 1.0 mg according to birth weight. In cases of severe coagulation defects caused by vitamin K antagonists, such as warfarin, higher doses of vitamin K supplements might be effective [47]. Sutor [49] reported the time required for recovery from coagulopathy by vitamin K administration in 4 infants with severe VKDB. In all infants, the PT was restored to 30%–50% of the normal value within 1 h of administering IV vitamin K (1–3 mg phylloquinone), with evident reduction in bleeding observed as early as 20 min [49,50]. A significant increase in the levels of all four vitamin K-dependent factors can be observed as early as 30 min after IV vitamin K administration, and within 2 h, the levels are typically within or near the lower limit of the normal range for neonates [11,49,50].

For severe bleeding episodes, it may be necessary to administer blood products, such as fresh frozen plasma (FFP) or prothrombin complex concentrate (PCC); vitamin K should not be administered [51,52]. PCC, which contains all four vitamin K-dependent factors, can rapidly reverse a vitamin K-dependent coagulopathy with a considerably lower volumetric load. Although there are no data on the dosage for the use of PCC in VKDB, a study conducted in adults has suggested a dose of 50 units/kg [51]. As per the abovementioned Japanese guidelines, an infusion of FFP (10–15 mL/kg) or PCC (50–100 units/kg) should be considered in severe cases and for very-low-birth-weight infants who cannot sufficiently utilize vitamin K due to immature liver function. Additionally, the use of recombinant factor VIIa has been reported for the treatment of severe intracranial hemorrhage in 3 infants with VKDB who required immediate surgical intervention [50,53].

Therefore, if there are any bleeding events due to suspected VKDB in an infant, IV vitamin K should be administered while awaiting blood product preparation and confirmation of the diagnosis based on laboratory results. If venous access cannot be established, vitamin K can be subcutaneously administered; however, it should not be intramuscularly administered in the presence of an existing coagulopathy.

## 5. Prevention of VKDB in Infancy and Prophylaxis Guidelines

To prevent early-onset VKDB, mothers receiving medications that impair vitamin K metabolism (excluding warfarin) should be administered vitamin K before delivery [54]. As per the Japanese guidelines, pregnant women using drugs that could impair the absorption of vitamin K (excluding warfarin) should be administered 15–30 mg vitamin K daily 2 to 4 weeks before delivery or their baby should receive 0.5–1.0 mg IV vitamin K_2_ administration [48]. However, a systematic review of the literature on antiepileptic drug use in pregnancy revealed that there was insufficient evidence to support vitamin K supplementation in the last weeks of pregnancy to reduce the risk of VKDB [55]. Further studies are required to establish a prophylactic strategy to prevent early-onset VKDB [56].

Classic VKDB rarely occurs in newborns who received parenteral vitamin K at birth. Two clinical trials conducted in the 1960s compared several doses of IM vitamin K supplements without prophylaxis on the incidence of classic VKDB [57,58]. Their results clearly demonstrated that vitamin K prophylaxis effectively reduces the incidence of classical VKDB without any severe adverse events [57,58].

Vitamin K prophylaxis (vitamin K_1_ 1 mg) via IM injection has been used worldwide. Conversely, peroral (PO) vitamin K administration became more widespread after Golding et al. reported an increased risk of childhood cancer with IM vitamin K administration [59]. Two studies compared the relative risk for late-onset VKDB between PO and IM vitamin K administration [60,61]. PO vitamin K administration appeared less effective with higher failure rates than IM vitamin K administration [6,62]. The Canadian Pediatric Society recommends that healthcare providers should counsel parents on the serious risk of VKDB if parents decline IM administration [63]. However, if some parents refuse IM vitamin K prophylaxis, PO vitamin K administration might be more acceptable. Cochrane reviews and systematic reviews have shown that there were no significant differences in coagulation status between PO and IM vitamin K administration [64]. However, the success of oral prophylaxis depends on the protocol regimen and compliance. In a nationwide prospective observational study conducted between 1998 and 2001 in Japan, six cases with classical VKDB were reported in which vitamin K was not administered at birth.

No clinical trials have been conducted to evaluate the effect of vitamin K prophylaxis on late-onset VKDB. However, epidemiological studies from various countries have suggested that the incidence of late-onset VKDB has significantly been reduced owing to the implementation of vitamin K prophylaxis programs [47,62,65,66] (Table 4.). PO vitamin K administration has been the primary method of prophylaxis in several countries, and the incidence of late-onset VKDB has varied: 1.6 per 100,000 infants in the UK [67], 1.9 in Japan [68], 5.1 in Sweden [69], and 6.4 in Switzerland [70] (Table 6). Moreover, some of these infants may have had underlying disorders that affected vitamin K metabolism and malabsorption. Unfortunately, these diseases, particularly cholestatic liver disease, often become apparent after the occurrence of VKDB. Therefore, a prophylactic regimen would be a preventative strategy for all infants, including those with unrecognized diseases, until diagnosis.

Sutor summarized the incidence of late-onset VKDB for several prophylactic strategies [39]. He reported that a regimen of 3 oral doses of vitamin K protects several infants (incidence of 0.44 per 100,000 infants), and there were no late-onset VKDB cases observed with 25 μg daily or 1 mg weekly vitamin K prophylaxis regimens [49]. However, a repeated PO regimen may not be practical due to lower patient compliance. One epidemiological study [6] conducted in Australia, Germany, the Netherlands, and Switzerland confirmed that 3 doses of 1 mg PO vitamin K supplement was less effective than IM vitamin K administration in neonates, although a daily PO dose of 25 μg (from weeks 1 to 13) after an initial PO dose of 1 mg may show similar efficacy. Recently, a 6-fold increase in daily oral vitamin K prophylaxis (from 25 to 150 μg) was reportedly associated with a significant reduction in the incidence of intracranial VKDB (from 3.1 to 1.2 per 100,000 live births) in the Netherlands [71]. This finding indicates that the increased prophylaxis volume (from a total of 0.175 to 1.05 mg/week) of vitamin K supplements reduces intracranial hemorrhage due to VKDB but is insufficient to completely prevent late-onset VKDB. Therefore, another factor other than dosage must be addressed to improve the efficacy of the prophylactic protocol.

A study from Denmark reported that weekly PO vitamin K supplementation for infants until they reached 3 months of age reduced the incidence of late-onset VKDB compared with a single PO dose [72]. Moreover, the report showed that prophylaxis was regarded as complete if the infant received at least 9 doses [64]. Therefore, adherence and instances of incomplete regimens (e.g., vomiting after oral administration and forgetting to take the vitamin K products) were not considered crucial in a weekly protocol regimen.

A total of five nationwide surveys on VKDB were conducted in Japan over a period of 20 years from 1981 to 2004 [68]. The introduction of PO prophylaxis in Japan with 1–3 PO doses of a syrup formulation of MK-4 (2 mg) substantially reduced late-onset VKDB. Approximately 4-fold reduction in the incidence of VKDB was observed from 10.5 per 100,000 live births in the first survey to 2.8 per 100,000 (95% CI 2.0 to 3.8) by 1988 (third survey) and further to 1.9 per 100,000 live births (1.2 to 3.0) by the fifth survey [66,68]. Moreover, no cases of late-onset VKDB were reported in one-third of infants who received 3 PO doses [73]. These findings suggested that this 3-dose PO regimen with MK-4 was similar in efficacy to parenteral prophylaxis, although few cases of failure have been reported. However, in the fifth nationwide surveillance conducted in Japan between 1999 and 2004, 71 late-onset VKDB cases, including 21 idiopathic cases, were reported [68]. Therefore, new guidelines were proposed in 2011 [48], and recently, the regimen with weekly PO vitamin K supplementation for infants until they reach 3 months of age has spread widely throughout Japan. Previously, no cases of late-onset VKDB in which the weekly PO vitamin K prophylaxis regimen was completed have been reported without any adverse effects, although a nationwide survey has not been conducted in Japan.

Preterm infants are at a higher risk for VKDB than term infants due to hepatic immaturity, delayed gut colonization with microflora, and other factors [74]. Several studies have revealed the differences in vitamin K-dependent coagulation factor levels in both term and preterm infants [7], and the serum levels of factors II, VII, IX, and X in term infants at birth were at approximately 50% of these levels in adults. Conversely, preterm infants have lower levels than term infants [75]. This difference might be caused by the coexisting delay of feeding, subsequently delayed microflora colonization of the gastrointestinal tract, and repeated exposure to antibiotics that prevent this colonization.

Recommendations for vitamin K prophylaxis at birth for preterm infants widely vary in terms of dosage and routes of administration; however, there is inadequate evidence to support any one clinical practice [7]. Some centers administer IV vitamin K supplements to preterm infants undergoing intensive care to avoid the pain and skin injury induced by IM. However, the efficacy of IV vitamin K administration differs from that of IM administration [76]. According to a study in preterm infants [77], a single 0.3 mg/kg dose of IV vitamin K supplement achieved similar plasma levels at 24 and 120 h to those achieved by PO or IM doses of 1.5 mg. A randomized clinical trial with a single vitamin K administration of 0.5 mg IM, 0.2 mg IM, or 0.2 mg IV at birth in 38 preterm infants showed that the serum vitamin K levels of all preterm infants were elevated above the normal range on day 5 but declined day 25, particularly in those who received IV vitamin K supplementation at birth [78]. Collectively, the circulating vitamin K levels following IV administration increase more rapidly but transiently than those after IM administration [79]; therefore, the IV injection should be repeated.

We recommend weekly PO vitamin K supplementation at 2 mg until 3 months of age based on the experiences in Denmark and Japan. Further national surveillance and studies are warranted to reveal the optimal prophylactic regimen in term and preterm infants for all forms of VKDB.

## Figures and Tables

**Table 1:** Vitamin K content (mg/L) of human, cow, and formula milk.

	*n*	Phylloquinone	Menaquinone-4
Human milk			
2–14 days	29	7.7 ± 4.1 (1.6–17.1)	2.7 ± 2.8 (<0.4–13.2)
15–60 days	76	9.2 ± 6.1 (1.6–33.9)	2.5 ± 3.0 (<0.4–16.2)
Cow’s milk	8	17.4 ± 3.0 (11.3–20.2)	4.5 ± 4.7 (<0.4–15.2)
Formula milk	12	19.9 ± 6.5 (12.6–32.0)	5.4 ± 4.6 (1.2–13.2)

Mean ± SD. Numbers in parentheses indicate a range.

**Table 2:** Physiological fecal levels of vitamin K (phylloquinone (K1) and menaquinones (MK-4–10)) in normal adults and neonates (ng/g dry weight).

		K_1_	MK-4	MK-5	MK-6	MK-7	MK-8	MK-9	MK-10
Adult	A	648	116	109	422	387	910	2054	11,854
	B	1898	289	281	2140	1988	511	3080	15,662
	C	5345	-	-	507	1826	2476	2702	7085
	D	1634	-	273	407	1071	525	-	5167
	E	2477	73	1570	189	753	389	1411	5745
	F	2220	537	1138	2071	2562	1273	2548	9585
Newborn	A	4	-	-	-	-	-	-	-
	B	18	-	-	-	1	-	-	-
	C	3	-	-	-	-	-	-	-
	D	10	-	-	-	-	-	-	-
	E	3	-	-	-	-	-	-	-
	F	2	-	-	-	1	-	-	-

**Table 3:** Possible factors inducing vitamin K deficiency in newborns.

1. Poor placental transfer of vitamin K
2. Immature gut flora
3. Low vitamin K content in breast milk and substantial differences among individuals
4. Poor intestinal absorption of vitamin K
5. Low activity level of vitamin K epoxide reductase

**Table 4:** Classification of vitamin K deficiency bleeding (VKDB) in infancy.

Classification	Time of Presentation	Etiology	Common Bleeding Sites
Early-onset VKDB	0–24 h	Maternal medications (e.g., warfarin and anticonvulsants)	Subperiosteal layer of the skull and intracranial, cranial, intrathoracic, and intra-abdominal regions
Classic VKDB	2–7 days	Mainly idiopathic, maternal medications, and breastfeeding	Gastrointestinal tract, nose, umbilical stump, and skin as well as at the wound after circumcision
Late-onset VKDB	2–12 weeks	Mainly secondary, underlying diseases (e.g., biliary atresia, cystic fibrosis, and other liver diseases with cholestasis), chronic diarrhea, occasionally idiopathic, and antibiotic therapy	Intracranial regions, skin, and the gastrointestinal tract

**Table 5:** Incidence of late-onset VKDB in different countries.

Country	Reference	Observation Period	Incidence Per 100,000 Infants (95% CI)
United Kingdom	34	1988–1990	4.4 (2.0–8.4)
Germany	35	1988–1998	7.2 (3.5–13.3)
Japan	36	1981–1983	10.5 (7.0–15.0)
Thailand	21	1981–1984	72.0

**Table 6:** Strategy of prophylaxis for vitamin K deficiency bleeding (VKDB) and incidence of late-onset VKDB in different countries.

Route	Country	Observation Period	Strategy of Vitamin K Prophylaxis	Incidence per 100,000 Infants Receiving the Regimen (95% CI)
IM	United Kingdom		1 mg at birth, 3 × 1 mg PO (day 1, week 1, and week 4)	0.1
	The United States		1 mg at birth	No data available
	Canada		1 mg at birth	0.37
	Denmark	2000–	2 mg at birth	No data available
	New Zealand		1 mg at birth	0.16 (0–0.46)
PO	The Netherlands	1992–1994	1 mg at birth, 25 μg/day for 13 weeks	0 (0–0.7)
		2005–2005	1 mg at birth, 25 μg/day for 13 weeks	3.2 (1.2–6.9)
		2014–2016	1 mg at birth, 150 μg/day for 13 weeks	1.2 (0.6–2.3)
	Germany	1993–1994	3 × 1 mg (days 1, 4–10, and 28–42)	1.3 (0.8–2.0)
		1995–1998	3 × 2 mg (days 1, 4–10, and 28–42)	0.4 (0.2–0.7)
		1997–2001	3 × 2 mg (days 1, 4–10, and 28–42)	0.8 (0.4–1.4)
		1997–2001	3 × 2 mg (days 1, 4–10, and 28–42)	0.44 (0.2–0.9)
	France		2 mg weekly for 6 months	No data available
	Australia	1993–1994	3 × 1 mg (days 1, 3–5, and 21–28)	1.5 (0.5–3.6)
	Denmark	1990–1992	1 mg at birth	4.5 (1.6–10.3)
		1992–2000	2 mg at birth, 1 mg weekly for 3 months	0 (0–0.9)
	Switzerland	1986–1988	1–3 mg at birth	6.4 (2.5–13.1)
		1995–2002	2 × 2 mg (days 1, 4)	1.2 (0–6.5)
		2003–present	3 × 2 mg (days 1, 4, week 4)	0.87 (0.24–2.24)
	Sweden	1987–1989	1–2 mg at birth	6.0 (3.7–9.8)
	United Kingdom		3 × 2 mg (days 1, 3–7, and week 4)	0.43
	Thailand	1988–1995	2 mg at birth	4.2–7.8
	Japan	1988–1990	3 × 2 mg MK-4 (days 1, 7, and 28)	2.8 (2.0–3.78)
		1994–2004	3 × 2 mg MK-4 (days 1, 7, and 28)	1.9 (1.2–3.0)

IM: intramuscular, PO: peroral, MK-4: menaquinone-4.
